# A New Methodology for Vibration Error Compensation of Optical Encoders

**DOI:** 10.3390/s120404918

**Published:** 2012-04-17

**Authors:** Jesus Lopez, Mariano Artes

**Affiliations:** Departamento de Mecanica, Universidad Nacional de Educacion a Distancia, C/Juan del Rosal, 12. Ciudad Universitaria, Madrid 28040, Spain; E-Mail: martes@ind.uned.es

**Keywords:** optical encoders, error compensation, vibration

## Abstract

Optical encoders are sensors based on grating interference patterns. Tolerances inherent to the manufacturing process can induce errors in the position accuracy as the measurement signals stand apart from the ideal conditions. In case the encoder is working under vibrations, the oscillating movement of the scanning head is registered by the encoder system as a displacement, introducing an error into the counter to be added up to graduation, system and installation errors. Behavior improvement can be based on different techniques trying to compensate the error from measurement signals processing. In this work a new “*ad hoc*” methodology is presented to compensate the error of the encoder when is working under the influence of vibration. The methodology is based on fitting techniques to the Lissajous figure of the deteriorated measurement signals and the use of a look up table, giving as a result a compensation procedure in which a higher accuracy of the sensor is obtained.

## 1. Introduction

Optical encoders are sensors used to measure the relative displacement between two mechanical parts. They are widely used in a vast range of applications, such as for example in robotics [1], tracking systems [2], machine tools [3] and everywhere high precision and resolution are required at a relatively low price. The measurement is accomplished by means of electrical signals generated in photodetectors because of the interference patterns between gratings. Ideal electrical measurement signals are composed of two quadrature signals of equal amplitude with zero mean value. Insofar that the encoder signals stand apart from these ideal conditions, errors are introduced in the measurement process with a consequent loss of accuracy of the sensor. Measurement signals deterioration can has its origin in different nature sources. Encoder errors can be classified primarily into those due to inaccuracies in the grating and those that arise when reading the grating. Both types of errors can have a systematic or variable nature. For example, the grating may have imperfections due to slit errors or by the presence of dust particles. In case of slit errors, the encoder line error is fixed or little changing in such a way that this error could be considered systematic in nature. This contrasts with the error variable in nature that appears when a certain localized area of the grating is affected by the presence of dust. An example of an error that may occur when reading the grating is that produced by non-parallelism of the gratings. It can be caused by system non-adjustment (constant error) or by straightness error of slides (variable over the travel length). As common accuracy and resolution grades are in micrometer and nanometer ranges, respectively, it is not hard to figure out that the performance of the encoder is sensitive to the margins of tolerances inherent to the manufacturing process. These errors have usually low values in contrast with those that arise when the encoder operates under certain solicitations that may occur when it is working. In case the encoder is working under vibrations, the errors can reach the accuracy of the sensor. This is mainly due to the fact that when the scanning head is vibrating in relation to the scale, the vibration movement is registered by the encoder system as a displacement and hence translated directly into the readout display, which increases and decreases continuously around a particular measure. This error superimposes to those mentioned above, giving as a result a significant detriment of the encoder's accuracy.

One approach to improve encoder's accuracy consists of analyzing the mechanical behavior of the sensor's components when they are facing to each one of these different nature solicitations. Under this approach, Alejandre and Artes [4–6] analyze encoder's components behavior in static, thermal and dynamic conditions. Recently, Lopez, Artes and Alejandre [7] analyze encoders' dynamic behavior using an improved version of the experimental technique described in the cited reference [6]. The improved technique is based on encoder's error estimation by means of fitting techniques to a discrete treatment of the Lissajous figure composed of the deteriorated measurement signals. As a result, a characterization of the error as a function of each one of the possible deteriorations is obtained. In another work [8], Lopez, Artes and Alejandre analyze possible causes of sources errors when the encoder is working under vibrations from a mechanical point of view.

Another point of view to improve the accuracy of the sensor is that just based on error compensation from signal processing techniques. Sensor's error compensation techniques based on signal processing constitutes a widely studied research field. The main reason for this lies on the fact that significant improvements of accuracy can be obtained normally at a lower cost than that associated to the introduction of mechanical modifications in the encoder design. There are several methodologies to accomplish encoder's error compensation [9]: Autocalibration, Look Up Tables (LUT), Filtering, Neural Networks [10], Wavelets and Blocking Amplifiers. Autocalibration and LUT techniques are of special interest for the purposes of the present work. Probably, the most referenced technique dealing with autocalibration is that proposed by Heydemann [11]. Basically, the problem is solved introducing in the measurement signals the parameters which produce the encoder metrological errors, arranging the equations in a least squares problem. More recently, Balemi [12] takes this method as a basis to formulate the problem with high computational efficiency to be implemented as an on-line error compensation technique by means of electronic signal processing components. Another point of view to accomplish the compensation has its basis on fitting techniques to the Lissajous figure composed of the deteriorated measurement signals. Lepple [13] studies the possibility of implementing this technique as an online interpolation procedure for encoders working at high speed. Fitting techniques have also been applied successfully to other position instruments such as interferometers [14]. This methodology contrasts with LUT techniques in the sense that the sensor does not need of any reference to increase its accuracy. Habitually, the elaboration of a LUT implies the use of a sensor of higher accuracy and resolution than the sensor that is going to be compensated [15]. The main advantage of LUT methodologies is that they are easily implemented as on-line procedures due to their high computational efficiency. A similar work close in aim and method to the cited paper [15] is that proposed by Boggarpu *et al.* [16], with the difference that no reference sensor is needed and the implementation can be done in a simple microcontroller rather than more specialized hardware. In this sense, the work developed by de Santiago-Perez *et al.* [17] remarks the special need for processing encoder's signals at a low computational cost while maintaining certain level of smoothness in the successive derivations of position up to jerk, in order to allow on-line implementation in obtaining the dynamics parameters from a machine-tool axis. This work is related in aim to that done by Morales-Vazquez *et al.* [18], but instead of being based on Daubechies Wavelet Transform with a fixed cut-off frequency it is proposed to use a high order FIR filter with an adaptive algorithm taking into account slow and fast movements in machining operations. Both works use the information available only from the encoder, without the need to use other sensors exclusively intended to measure speed or accelerations. Under this approach it is possible to obtain motion dynamics in terms of position, velocity, acceleration and jerk but it is not present the information of vibrations [1]. There are certain applications where it is needed to complement the information available from the encoder with other sensors giving way to what is known as fused sensors. Under this approach, in the cited reference [1] a triaxial accelerometer is used together with an encoder in order to be able to estimate vibrations and inclination on industrial manipulator robot links. The same primary sensors, an encoder and an accelerometer, are used in [19] applying Kalman filters for the assessment of forward kinematics of an industrial robot. In another work [20], the information available from an accelerometer is complemented by the current output of a servoamplifier in order to improve the online quantitative estimation of flank-wear area in CNC machine inserts.

When the encoder is operating under vibrations there are certain particularities associated with the deterioration of the measurement signals that can be treated with “*ad hoc*” procedures. In this work a novel methodology to compensate encoder error under sine vibration is presented and discussed. The methodology is based on fitting and LUT techniques from the information available of the encoder itself and an accelerometer.

## 2. Encoder Errors

### 2.1. Metrological Errors of Optical Linear Encoders

A mathematical description of the encoder measurement signals is given by:

(1) $$\begin{array}{c} S_{A}(x)=B_{1}+A_{1}F_{1}\left(2\pi \frac{x}{p}+\Phi_{1}\right)\\ S_{B}(x)=B_{2}+A_{2}F_{2}\left(2\pi \frac{x}{p}+\Phi_{2}\right) \end{array}$$

where *A*_1_ and *A*_2_ are the amplitudes, *B*_1_ and *B*_2_ the signal's mean values, *Φ*_1_ and *Φ*_2_ the signal phases, *F*_1_ and *F*_2_ are functions that represent the shape of the signals, *x* is the position measured and *p* is the grating period of the encoder. Ideal conditions are given by:

(2) $$\begin{array}{c} A_{1}=A_{2}\\ B_{1}=B_{2}=0\\ \Phi_{1}=0;\Phi_{2}=\frac{\pi }{2}\\ F_{1}=F_{2}=\text{sine functions} \end{array}$$

and position calculation is done by means of the well-known arctangent algorithm [21]:

(3) $$x=\frac{p}{2\pi }\text{arctan}\left(\frac{S_{A}}{S_{B}}\right)$$

If the above conditions are not satisfied metrological errors appear with a consequent detriment of encoder's accuracy. Sanchez-Brea and Morlanes [22] use this description to present a methodology to estimate the error committed by the encoder for each one of the particular cases of the conditions represented by Equation (2). Defining $f=\frac{x}{p}$ as the fundamental frequency of the measurement signals, by means of linear series expansion they obtain:

(4) $$\begin{array}{c} u_{\text{off}}(t)=\frac{p}{2\pi }\cos(2\pi ft)\frac{\Delta B_{1}}{A}\\ u_{\text{amp}}(t)=\frac{p}{4\pi }\sin(4\pi ft)\frac{\Delta A_{1}}{A}\\ u_{\text{ph}}(t)=\frac{p}{2\pi }\cos^{2}(2\pi ft)\Delta \Phi_{1} \end{array}$$

where *u*_off_is the error due to non-zero mean values; *u*_amp_ is the relative amplitude error and *u*_ph_, represents the error committed by the encoder when the phase between them is not exactly π/2. In case of non-zero mean values the Lissajous figure is not centered at the ideal (0,0) location and the error has the same frequency value of the measurement signals. In case differences between signals amplitudes exist, the Lissajous figure becomes an ellipse with the major axis at 0 or π/2 depending on if amplitude of signal *S_A_* is higher than the amplitude of signal *S_B_* or viceversa, respectively. In case of phase error, it is introduced in the error a systematic component due to the quadratic term and the Lissajous figure becomes an oval with the axis at π/4. In these two last cases the frequency of the error is double to that of the measurement signals frequency. Another type of error refers to the shape of the signal, but most of the times this error is negligible and it is not considered in the present work. Additionally, when the encoder is operating under vibration, there is a relative movement between the gratings that introduces a measurement error at the frequency of the excitation, as it is explained in the next section.

### 2.2. Encoder Error Due to Vibration

**Figure 1.**
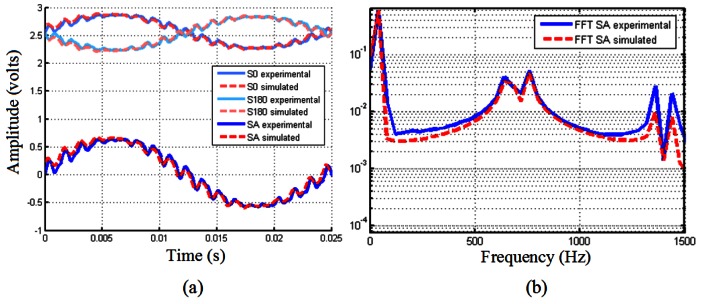
(**a**) Experimental signals *S*_0_ and *S*_180_ generated in the photodetectors with resultant measurement signal *S_A_* and simulation by means of Equation (5); (**b**) FFT of the experimental and simulated measurement signal *S_A_*.

Figure 1(a) shows the signals *S*_0_ and *S*_180_ generated in the photodetectors of a four field scanning encoder and the resultant measurement signal *S_A_*(=*S*_0_ − *S*_180_) deteriorated because of the presence of a 700 Hz and 100 ms^−2^ sine vibration at the same time the encoder is working. For its generation, a 20 μm grating period encoder has been operated on an electrodyamic shaker. In this figure a harmonic superimposed to the measurement signals can be appreciated with a frequency equal to that of the excitation frequency. As it can be observed the amplitude of the harmonic is minimum when the photodetectors are entirely illuminated or minimally illuminated, *i.e.*, when the lines of one of the gratings coincide with the lines or with the gaps of the other. On the other hand, when the gratings are partially superimposed the amplitude of the vibration harmonic reaches higher values. This can be easily explained thinking of the phenomenon as two independent movements going together. One movement would be that of the normal operation of the encoder, in which one of the gratings moves always forward with respect to the other grating, giving as a result a measurement signal with a frequency determined by the velocity of operation of the encoder. The other movement would be the same grating moving backward and forward repetitively with a certain displacement that depends on the frequency and severity of the vibration. This way, the first movement determines the centered relative position between the gratings at which the second movement takes place. If the lines of one of the gratings coincide with the lines or with the gaps of the other grating, this second movement will result in an alternating movement where the lines are partially coincident thus producing a minimum variation of light intensity in the photodetectors. On the other hand, if the lines of the two gratings are initially overlapping, the two utmost positions of the oscillating movement will be near the situations where the grating lines of one the gratings are coincident with the other grating's lines or with the gaps, hence producing a maximum variation of light intensity giving as a result maximum amplitude of the vibration harmonic. Figure 1(b) shows the FFT of this deteriorated encoder's measurement signal. The first peak corresponds to the velocity of 0.8 mm/s (40 Hz) of operation of the encoder and at the frequency of the vibration excitation (700 Hz) it can be appreciated that there is a doublet instead of a single peak. This doublet is a consequence of the fact that the vibration harmonic is added to the main signal when the grating moves from a situation where the photodetector is minimally illuminated towards a maximum light intensity condition, *i.e.*, the utmost movement of the vibrating grating produces light intensity increments. On the other hand, when the vibrating grating is moving towards a minimum light intensity condition from the fully illuminated photodetector, the vibration harmonic is subtracted from the main signal. Only from a frequency domain point of view, this can be seen as if the phase of the vibration harmonic is increased by a half cycle each time the light intensity reaches a maximum or a minimum. Taking these considerations into account it is possible to approximate mathematically the effect of vibration on the measurement signals generated in the photodetectors as follows:

(5) $$\begin{array}{c} S_{0,\text{vib}}\left(0<t\leq \frac{T}{4}\right)=S_{0}+\delta \left|\text{sinc}\left(\sin\frac{1}{2}(2\pi f_{\text{exc}}t+\emptyset )\right)\cos(2\pi ft)\right|\\ S_{0,\text{vib}}\left((2i-1)\frac{T}{4}<t\leq (2i+1)\frac{T}{4}\right)=S_{0}+{\left(-1\right)}^{i}\delta \left|\text{sinc}\left(\sin\frac{1}{2}(2\pi f_{\text{exc}}t+\emptyset )\right)\cos(2\pi ft)\right| \end{array}$$

where *T* is one period of the signal generated in the photodetector; *f*_exc_ is the frequency of the vibration excitation and *f* is the fundamental frequency determined by the velocity of the encoder. The normalized cardinal sine function (sinc) applied to the vibration harmonic adapts quite well to the effect of vibration, as can be seen in the simulated signals of Figure 1. Taking into account the amplitude modulation introduced by the cosine term at the velocity of the encoder (*f*), we obtain as a result a signal that varies between 0 and 1 (it has to be indicated with the absolute value symbol | |, with the maxima corresponding to the situations where the grating lines are half superimposed, and the minima corresponding to the maximum or minimum light intensity situations. It is necessary to introduce the term 1/2 because the normalization of the sinc function doubles the frequency value of the original signal. The term Ø is the phase between the vibration harmonic and the measurement signal. The phases associated to the harmonics that form the doublet can be almost equal or differ in practically half a cycle from the pure vibration harmonic sine at 700 Hz. The issue concerning which one to choose is not a trivial matter and will be discussed in the next section. Another advantage of the mathematical description used is that having a signal that varies between 0 and 1 the amplitude factor *δ* will be directly related to the amplitude of the measurement signal, as the output of most of the analog encoders commercially available is 1 volt peak to peak. Determination of an appropriate value for *δ* is complex, due to the nature of the vibration movement. As we have said above, we can think of the whole process as two independent movements. We say independent because the error due to vibration will depend directly to what extent one grating moves in the alternating movement relative to the other, and this movement depends on the accelerations that are acting on the encoder and on its mechanical design. Here is the keypoint of the methodology proposed as, with the procedure described in references [6,7], it is possible to estimate the relative movement between the gratings for a given range of frequency excitations, allowing to compensate the error due to vibrations effectively.

## 3. Methodology for Error Compensation

The proposed methodology is based on the combined use of fitting techniques to the Lissajous figure and the use of LUT. Basically, the methodology consists of the estimation of each one the types of errors separately just to build a compensation signal that will be composed of the addition of the individual error contributions. The process is tackled in two steps. In the first step the metrological errors of the encoder due only to manufacturing tolerances are estimated. The second step deals with the compensation of the error due to vibrations.

### 3.1. Estimating the Contribution of Metrological Errors to the Total Error

**Figure 2.**
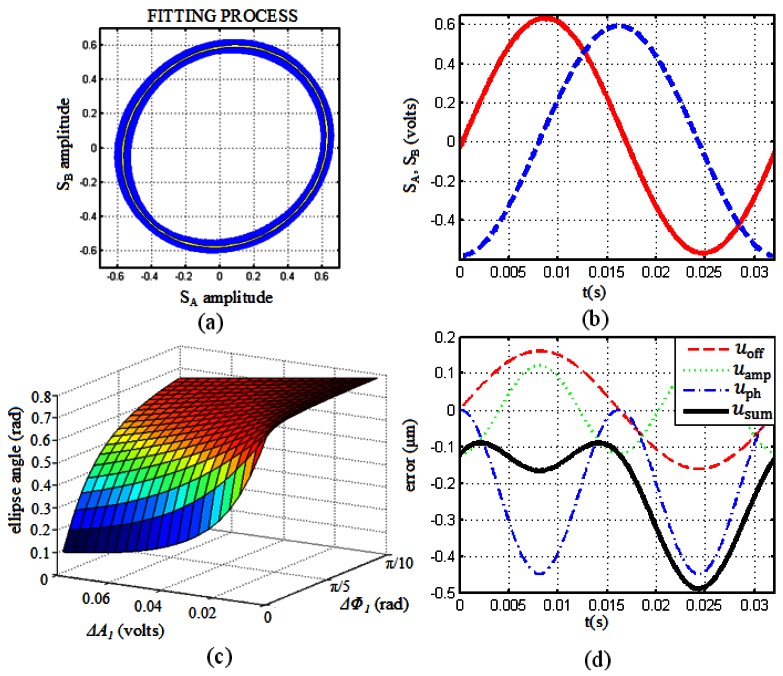
(**a**) Lissajous figure for the whole measuring length under normal operating conditions (dotted-blue) and best fitting ellipse (continuous-white); (**b**) Example of only period for the measurement signals represented in Figure 2(a) (SA: continuous-red; SB: dashed-blue); (**c**) Interpolating surface for the estimation of the phase error term Δ*Φ*_1_; (**d**) offset error (dashed-red), amplitude error (dotted-green), phase error (dashed-dotted blue) and the sum of all of them (so-called metrological errors, continuous-black).

For the generation of each one of the contributions to the compensation signal of the so-called metrological errors the use of the group of Equation (4) is proposed. The estimation of each one of the terms that appear in these equations can be accomplished by means of fitting techniques to the Lissajous figure when the encoder is operating without the presence of vibrations. Figure 2(a) represents the Lissajous figure of the encoder for the whole travel length (dotted-blue). A certain point dispersion that produces a line width due to variations in amplitudes, offsets and phases over the entire travel length of the sensor has to be noted. Once this figure is obtained, the ellipse that produces the best fit [23] to the whole set of points is calculated (represented in continuous-white in Figure 2(a)). Now we are ready to estimate the metrological errors of the encoder. The estimation made in this manner will produce mean values of the metrological errors, just a set of fixed values that will be the best representation for the whole measuring length of the encoder.

From the ellipse parameters the term Δ*B*_1_ can be estimated as $\sqrt{C_{x}^{2}+C_{y}^{2}}$, where *C_x_* and *C_y_* are the center components of the ellipse obtained. To calculate the contribution to the total error due to differences in relative amplitudes, the term Δ*A*_1_ has been estimated as $\sqrt{{\left(R_{x}-A\right)}^{2}+{\left(R_{y}-A\right)}^{2}}$, where *R_x_* and *R_y_* are the radii of the ellipse obtained and *A* is the ideal amplitude of the measurement signals, gotten from the ideal circle. The estimation of the term Δ*Φ*_1_ just from the fitting process is a bit more difficult process. The difficulty lies on the fact that differences of relative amplitudes tend to rotate the major axis of the ellipse to 0 radians while the phase error tends to rotate it to π/4 radians. Because both amplitude and phase errors are present in this case, the resulting rotation of the ellipse would be just a combination of the two types of errors. Through the estimation of the maximum and minimum values that these error terms can have for this encoder, an interpolating surface is determined (Figure 2(c)). Here, all the possible values of the ellipse angle that correspond to each one of the combinations considered for amplitude and phase errors are registered. Knowing the actual angle value of the ellipse obtained and the amplitude error term it is immediate to calculate the phase error term Δ*Φ*_1_ from the interpolating surface. Once all the error terms have been estimated, the offset, the amplitude and the phase errors are generated according to the set of Equation (4), taking into account the frequency relations between these errors and the fundamental operational frequency of the encoder, which can be obtained from the FFT of the measurement signals.

It has to be taken into account that due to the fitting process itself and the use of an interpolation surface of limited resolution, the reconstruction of the error terms in this way will be approximate. However, as most of the error contribution is at the fundamental frequency and the first harmonic, typical error values associated with the reconstruction are around 5%, and less than 10% in any case.

### 3.2. Estimating the Contribution of Vibration Error to the Total Error

**Figure 3.**
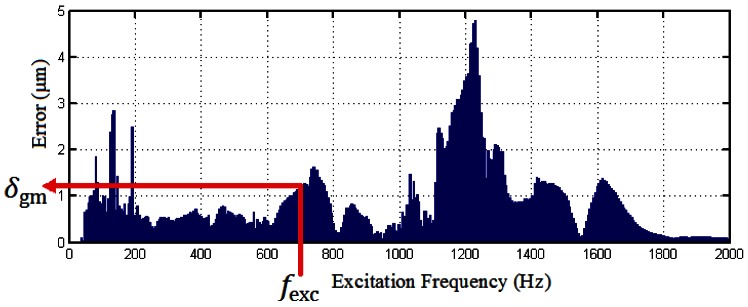
Error chart that can be used as a LUT to construct the vibration compensation signal.

A second step consists of the error estimation due to vibrations that produce the relative movement of the gratings. To accomplish this task it is necessary to previously elaborate a look up table (LUT) by means of the procedure described in the references [6,7]. In this procedure, the encoder is fixed to an electrodynamic shaker to undergo a sine frequency sweep registering the encoder's measurement signals. Previously, it is necessary to calculate the acceleration which the encoder is under, just to set the parameters that determine the severity of the test. The encoder is fixed. Therefore no error conditions are represented as a point in the Lissajous figure. If for a particular frequency value a resonance appears, the gratings would start moving relatively to each other, becoming the Lissajous figure an arc. To estimate the error due to the relative movement of the gratings, the measurement signals registered are processed taking a certain amount of samples directly related to frequency resolution desired in the elaboration of the look up table. Calculating the parameters of the conic that produces the best fit to the arc, it is possible to calculate the error due only to the relative movement of the gratings removing the offset, amplitude and phase errors. This way, estimating the angle covered by the Lissajous figure at a particular frequency is possible to obtain a look up table as that depicted in Figure 3. This chart is the mean of ten experimental measurements done with the experimental procedure described. Test's severity parameters have been a sine sweep from 20 Hz to 2,000 Hz at a rate of 22 Hz/s with amplitude of 100 ms^−2^.

Taking the look up table as a basis, it is proposed to build a signal to compensate the error due to vibrations as follows:

(6) $$u_{\text{vib}}=M_{\text{me}}+\frac{1}{2}\delta_{\text{gm}}\sin(2\pi f_{\text{exc}}t+\emptyset )$$

The above formula is based on the assumption that the error due to the relative movements between the gratings is an error that is superimposed to those so-called metrological errors. This way, *M*_me_ is the mean of the metrological errors. The mean value of metrological errors is due to the imperfect phase conditions as it is the only one that produces a systematic component. The term *δ*_gm_ represents the amplitude of the error due to the relative movement of the gratings, which is obtained reading in the LUT of Figure 3. To do this, first of all it is necessary to calculate the frequency of the vibration excitation, *f*_exc_, that can be determined from the harmonics of the FFT analysis of the measurement signals previously done. There is an uncertainty associated with this calculation due to the presence of the doublet as we have said in Section 2. Knowing that the doublet is nearly centered at the excitation frequency, it is possible to estimate *f*_exc_ from the frequencies of both harmonics that form the doublet. Once these frequencies are determined it is also needed to calculate the phases associated to them, in order to establish the term Ø. As it has been stated in Section 2.2 the phases associated to the harmonics that form the doublet are almost equal or differ in practically half a cycle of the pure vibration harmonic sine at 700 Hz. In case that both phases differ, it is possible to compensate the error with both values, but if it is chosen the minimum phase the compensation signal (the sum of all the error contributions) has to be added to the position calculated by the arctangent algorithm, and subtracted otherwise. However, there is always some residual error after the compensation and to minimize the systematic component in this residual error certain considerations regarding the term Δ*Φ*_1_ have to be made. They can be summarized as follows:

(7) $$\begin{array}{c} \emptyset =\min(\emptyset_{1},\emptyset_{2})\text{if}\Delta \Phi_{1}<0(\text{the compensation signal}\;S_{\text{comp}}\text{has to be added to the position})\\ \emptyset =\min(\emptyset_{1},\emptyset_{2})\text{if}\Delta \Phi_{1}>0(\text{the compensation signal}\;S_{\text{comp}}\text{has to be substracted to the position}) \end{array}$$

where Ø_1_and Ø_1_ are the phases associated to the first and second peak that form the doublet at the excitation frequency; Ø is the phase associated to vibration error term *u*_vib_ (Equation (6)); and Δ*Φ*_1_ is the increment or decrease in phase between the measurement signals with relation to the ideal 90° degrees (Equation (4)), calculated by the process described in Section 3.1. It has to be taken into account that the value of the error obtained in the chart constitutes the maximum amplitude of an oscillation movement around of ideal position that, as the encoder has been tested at a fixed position, it has to be zero. This way, the term 1/2 is introduced to consider the relative movement between gratings as peak to peak amplitude. Once the error due to vibrations is estimated by the procedure described, it is only needed to add up this error term to those metrological errors to obtain the compensation signal:

$$S_{\text{comp}}=u_{\text{off}}+u_{\text{amp}}+u_{\text{ph}}+u_{\text{vib}}$$

### 3.3. Methodology for Vibration Error Compensation

In order to summarize and present the methodology in a clear manner for practical purposes, in this section the several steps needed to accomplish the whole procedure are listed as follows:

1. Operate the encoder for the whole measuring length registering the sensor's measurement signals with the source of vibration (for example, the machine in which the sensor is installed) turned off and calculate the ellipse that best fits the whole set of points that it is obtained when plotting both amplitudes of the measurement signals against each other. From this ellipse, compute Δ*B*_1_, Δ*A*_1_ and Δ*Φ*_1_ as explained in Section 3.1. The values obtained will be always the same values for the generation of the compensation signal.
2. Place the encoder in the shaker and set the acceleration level, frequency range of interest and sweep rate (frequency resolution wanted) to elaborate a look up table by means of the procedure described in references [6,7]. Note that several LUT can be elaborated using the more common accelerations and frequency ranges.
3. Turn on the machine or source of vibration that affects the performance of the encoder and put an accelerometer next on the machine and obtain the amplitude of acceleration and the frequencies at which the machine operates.
4. From the FFT of one of the measurement signals compute *f*, *f*_exc_, Ø and with *f*_exc_ using the look up table of point 2 to calculate *δ*_gm_ by linear interpolation. Compute *u*_off_, *u*_amp_, *u*_ph_ by the set of Equation (4) and calculate the mean of this signal to compute *M*_me_ and *u*_vib_ by Equation (6). Then add it to the metrological errors to construct the compensation signal *S_comp_* = *u*_off_ + *u*_amp_ + *u*_ph_ + *u*_vib_. Finally, add or subtract the compensation signal to the position determined by the arctangent algorithm applied to the deteriorated measurement signals following the criteria exposed by Equation (7).

Steps 1 and 2 and software to compute Step 4 could be developed by the encoder manufacturer. The Step 3 would be the only one that would be done in the industrial environment to obtain the acceleration and frequencies at which the machine operates. It has to be noted that in the methodology described no use of a sensor of higher accuracy or resolution has been necessary.

Finally, for the sake of clarity the methodology has been exposed considering that the vibration excitation is produced mainly in one direction, but the performance of the encoder can be different regarding the direction of vibration, as other modes of vibration of the encoder's components can be excited. This case is contemplated in the referenced work [8], providing several LUTs like that depicted in Figure 3 for each one of the possible positions of the encoder in relation to the vibration direction. For the general case where there is no one dominant direction of the excitation, a combination of the values of each one of the LUTs in the different directions is recommended. The combination can be done proportional to the relative level of accelerations detected by the accelerometer between each one of the axis considered.

## 4. Experimental Results

**Figure 4.**
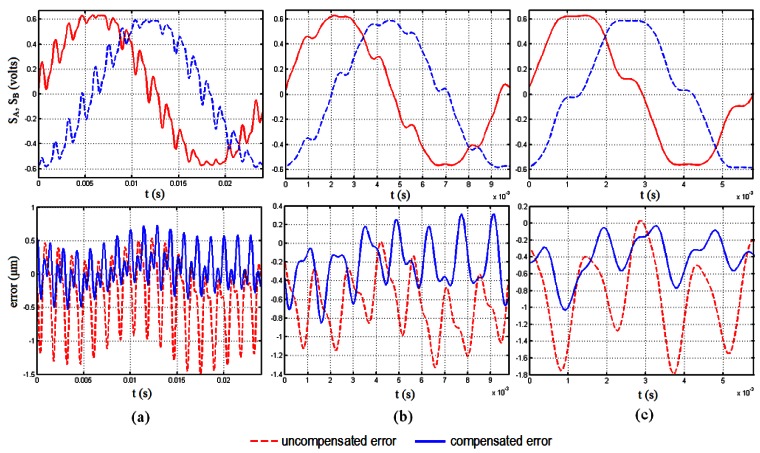
Error compensation results. Constant excitation frequency (700 Hz) and increasing speed operation of the encoder (0.8 mm/s (**a**), 2 mm/s (**b**), 3.4 mm/s (**c**)); (dashed-red: uncompensated error; continuous-blue: compensated error).

**Figure 5.**
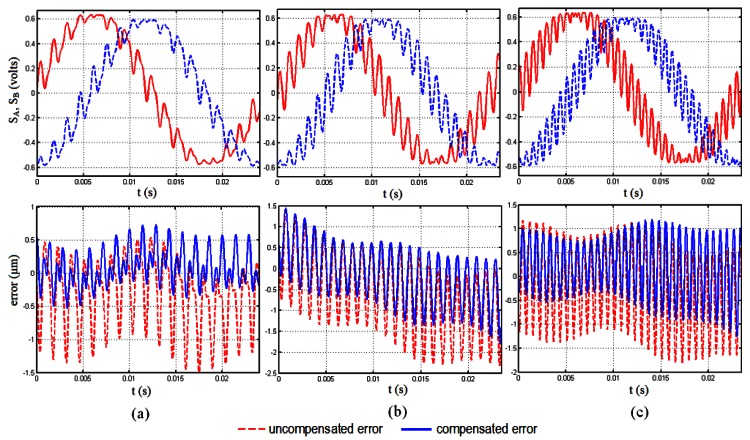
Error compensation results. Increasing excitation frequency (700 Hz (**a**), 1,000 Hz (**b**), 1,400 Hz (**c**)) and constant speed operation of the encoder (0.8 mm/s); (dashed-red: uncompensated error; continuous-blue: compensated error).

Results regarding the application of the above methodology are presented in Figures 4 and 5. For all cases the amplitude of the vibration excitation is just the same as that used for elaboration of the LUT (100 ms^−2^). Concretely, Figure 4 presents the error compensation results obtained in the case where the encoder is operating with different speeds while the vibration excitation frequency is maintained constant. The opposite case has been considered for the study presented in Figure 5, *i.e.*, when the encoder is operating at constant speed and is subjected to excitations that vary in frequency. The encoder under study is the model SP70 from Fagor Automation, a sealed incremental sinusoidal type with accuracy of 5 μm and 20 μm of grating period. For the vibration generation an electrodynamic shaker (TIRAvib 50101) has been used. The vibration reference is provided by means of an accelerometer (Brüel & Kjaer Type 4383) to maintain the level of the vibration excitation, used in a closed loop control with controller Vibration Research VR8500. The encoder's measurement signals have been registered by the Time Data Recorder Module of the data acquisition system PULSE (Brüel & Kjaer). A sampling frequency of 16,384 Hz has been chosen, almost one hundred times higher than the maximum frequency corresponding to the velocity of the encoder considered (174 Hz), and more than ten times higher than the maximum frequency of the different vibration excitations (1,400 Hz), in order to have a faithful representation of the measurement signals to be processed later in MATLAB.

In Figure 4(a) the encoder is operating at a speed of 0.8 mm/s (40 Hz) and is subjected to a sine vibration excitation of 700 Hz. In this figure, it can be observed how the maximum and minimum errors at which the error oscillates are considerably reduced, as the encoder uncompensated error oscillates at a maximum value of 1.5 μm and is reduced to a maximum rounding 0.7 μm. In case the frequency excitation is maintained constant and the speed of the encoder is increased the algorithm works well as it can be observed in Figure 4(b,c). This case can be seen as if the fundamental frequency of the measurement signals and that of the harmonic corresponding to the vibration excitation are getting closer in the spectrum. In Figure 4(b) the speed of the encoder has been increased to 2 mm/s (100 Hz) and the compensated error shows values below those of the uncompensated case for the whole period of the measurement signals. The better performance is clear in case the speed is increased again until a value of 3.4 mm/s (174 Hz), represented in Figure 4(c). In this figure it can be appreciated how the maximum error is reduced from a value of 1.8 μm to a maximum peak of 1.0 μm, giving error values significantly lowers than the uncompensated case.

**Figure 6.**
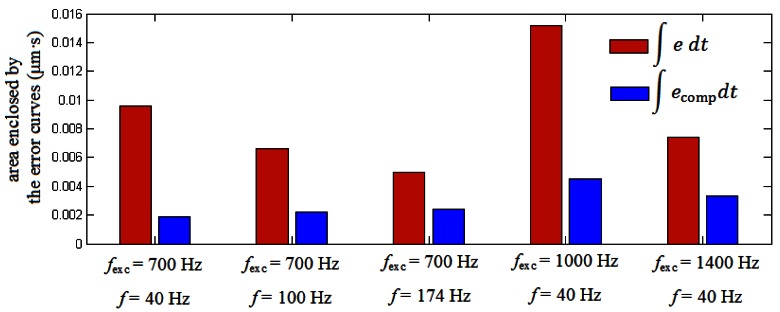
Area enclosed by the error curves before and after the compensation.

Figure 5 shows the results of the application of the algorithm when the encoder operates at a constant speed of 0.8 mm/s (40 Hz) subjected to excitation frequencies of 700 Hz (Figure 5(a)), 1,000 Hz (Figure 5(b)) and 1,400 Hz (Figure 5(c)). In these figures it can be appreciated that again the algorithm reduces the error committed by the encoder effectively, although the performance is not as good as in the case of increasing the velocity of the encoder instead the frequency of the excitation. This is due to the uncertainty inherent to the determination of the frequency excitation parameter, *f*_exc_, from the FFT of the measurement signals. This gives as a result that, if frequencies of the error and from the compensation signal do not match exactly, a certain phase is introduced between both, making the algorithm get worse as the encoder travels along the measuring length. Despite this, it is possible to reduce the error from a value of 2.3 μm to 1.3 μm for some peaks in case of 1,000 Hz of excitation frequency and from 1.8 μm to 0.9 μm in case of 1,400 Hz of excitation frequency. Instead of analyzing the performance of the method for single peaks it is more interesting to have an idea about how it performs for the whole period considered. From this point of view Figure 6 shows the area enclosed by the error curves before and after the compensation. We can see in this figure how the performance is significantly improved for all the cases considered. The best performance corresponds to the case of 700 Hz of excitation frequency with a velocity of the encoder of 0.8 mm/s, with an improvement of 80%. It has to be noticed that even for the worst cases there are still significant improvements or around 50% of the parameter considered when using the methodology proposed.

## 5. Conclusions

In this work a new methodology for sine vibration error compensation of optical linear encoders has been presented. When the encoder operates subject to vibrations certain particularities arise that allow using an “*ad hoc*” procedure. The procedure is based on the miscellaneous utilization of fitting techniques to the deteriorated Lissajous figure of the composed measurement signals and the use look up tables.

Results show that maximum and minimum errors at which the error oscillates are reduced considerably. When the encoder operates at a constant speed subjected to different excitations, it can be appreciated that the algorithm reduces the error committed by the encoder effectively, although the performance is not as good as in the case of increasing the velocity of the encoder instead the frequency of the excitation. We can conclude that the performance improves significantly for all the cases analyzed and it has to be noticed that even for the worst cases still there are significant improvements of around 50% for the parameter considered.

The proposed methodology allows an individualized treatment of the different types of errors that, when combined, permits one to obtain a compensation signal of similar shape and with similar values to the error that the encoder produces when it works under vibrations. The whole procedure is accomplished without the need to use a sensor of a higher accuracy or resolution. The simplicity of the different mathematical expressions used to calculate the different contributions to the final compensation signal together with the high computational efficiency of LUT procedures, makes the methodology suitable for use as an online procedure for compensating encoder error under sine vibration, once the error is characterized in a previous step off-line.
